# Solvency II solvency capital requirement for life insurance companies based on expected shortfall

**DOI:** 10.1007/s13385-017-0160-4

**Published:** 2017-10-14

**Authors:** Tim J. Boonen

**Affiliations:** Amsterdam School of Economics, University of Amsterdam, Roetersstraat 11, 1018 WB Amsterdam, The Netherlands

**Keywords:** Solvency II, Solvency capital requirement, Expected shortfall, Value-at-risk

## Abstract

This paper examines the consequences for a life annuity insurance company if the solvency II solvency capital requirements (SCR) are calibrated based on expected shortfall (ES) instead of value-at-risk (VaR). We focus on the risk modules of the SCRs for the three risk classes equity risk, interest rate risk and longevity risk. The stress scenarios are determined using the calibration method proposed by EIOPA in 2014. We apply the stress-scenarios for these three risk classes to a fictitious life annuity insurance company. We find that for EIOPA’s current quantile 99.5% of the VaR, the stress scenarios of the various risk classes based on ES are close to the stress scenarios based on VaR. Might EIOPA choose to calibrate the stress scenarios on a smaller quantile, the longevity SCR is relatively larger and the equity SCR is relatively smaller if ES is used instead of VaR. We derive the same conclusion if stress scenarios are determined with empirical stress scenarios.

## Introduction

Solvency II is the new supervisory framework that is in force from 2016 for insurers and reinsurers in Europe. It puts demands on the required economic capital, risk management, and reporting standards of insurance companies. Solvency II focuses on an enterprize risk management approach towards required capital standards. Its main objective is to ensure that insurance companies hold sufficient economic capital to protect the policyholders as it aims to reduce the risk that an insurer is unable to meet its financial claims (see, e.g., [17]). The required capital is based on the risks that the insurer faces. This capital requirement is called the solvency capital requirement (SCR) and covers all the risks that an insurer faces. EIOPA [25] defines the SCR of an insurance or reinsurance company as the value-at-risk (VaR) of the basic own funds subject to a confidence level of 99.5% on a 1-year period. In this paper, we base our model on the solvency II calibrations of EIOPA [25, 26].

The value-at-risk is a widely used risk measure and can be described as the maximum loss within a certain confidence level. In the case of the SCR, the confidence level of 99.5% tells us that the company can expect to lose no more than VaR in the next year with 99.5% confidence, so, on average, only once every 200 years the VaR loss level will be exceeded. The value-at-risk is however criticized for not being sub-additive [4]. Sub-additivity of a risk measure ensures that diversification is rewarded. Besides not being sub-additive, the VaR does also not consider the shape of the tail beyond the confidence level. This means that the VaR does not take into account what happens beyond the confidence level, so it does not consider the worst case scenarios. This is discussed by Acerbi and Tasche [3] and Yamai and Yoshiba [46]. The fact that VaR is not sub-additive and that it does not consider the tail beyond the confidence level might make it less suitable to calculate capital requirements.

Coherent risk measures are sub-additive, and consider the shape of the tail beyond the confidence level. A risk measure is called coherent if it satisfies the axioms translation invariance, sub-additivity, positive homogeneity, and monotonicity [4]. The most popular coherent risk measure is the expected shortfall (ES). This risk measure is equal to the expected value of the loss, given that the loss is larger than the value-at-risk, therefore the expected shortfall also depends on the quantile used. Two other regulatory frameworks for financial institutions, the Swiss Solvency Test and the Basel III framework, use the expected shortfall as risk measure.

The contribution of this paper is not to claim that the expected shortfall is a better risk measure than the value-at-risk. Instead, we exemplary analyze the effects on three main risk factors of the total SCR for a fictitious life annuity insurer if the solvency II SCR calibration is based on expected shortfall instead of value-at-risk. The use of expected shortfall for insurance stress testing is also suggested by CEIOPS [12], Sandström [40], and Wagner [45]. We hereby consider this hypothetical change in regulation for three major risk classes: equity risk, interest rate risk, and longevity risk. Moreover, we analyze what happens if all SCRs are determined via empirical stress scenarios.

The standard model of solvency II explicitly assumes a Gaussian distribution for returns in some risk classes. If a return is Gaussian, the value-at-risk, as prescribed by solvency II, and expected shortfall provide similar information (e.g., [46]). If returns are non-Gaussian, which is observed for the most important classes of risk, the use of value-at-risk might lead to a mismatch of the SCR and the underlying riskiness. In this paper, we examine what the effects are of using such a standard model when risks are calibrated using the expected shortfall instead. We find that for EIOPA’s current quantile 99.5% of the value-at-risk, the stress scenarios of the various risk classes based on expected shortfall are close to the stress scenarios based on value-at-risk. We show that if EIOPA aims to keep the solvency capital requirement the same if it would switch to v, it should set the confidence level at approximately 98.8%. So, applying a 99% confidence level for the expected shortfall leads to an increase in the solvency capital requirement. Moreover, might EIOPA choose to calibrate the stress scenarios on a smaller quantile, the longevity SCR is relatively large and the equity SCR is relatively small if expected shortfall is used instead of value-at-risk.

This paper is set out as follows. Section 2 defines the risk measures value-at-risk and expected shortfall. Section 3 introduces the part of the solvency II regulations that we use in this paper. Section 4 states our methodology, and shows the stress scenarios for the solvency capital requirements under the two risk measures. Section 5 shows the main results, and a sensitivity analysis. Section 6 illustrates our results in relations to other regulatory frameworks, and Sect. 7 concludes.

## Risk measures to calibrate solvency capital

Risk measures are used to determine the amount of economic capital to be kept in reserve in order to protect a company for any negative risky impacts that may arise in the future. A risk measure maps random variables into real numbers. In this paper we discuss two well known risk measures: the value-at-risk (VaR) and the expected shortfall (ES), where we refer to McNeil et al. [36] for more details about these two risk measures.

The value-at-risk with confidence level $\alpha \in (0,1)$ is the $\alpha$-quantile, i.e.,

1 $$\begin{aligned} \textit{VaR}_\alpha (X) = \inf \{x \in \mathrm{I} \negthinspace \mathrm{R}:\mathbb {P}(X > x) \le 1-\alpha \}, \end{aligned}$$

for all random variables *X* (a loss).

Coherence of risk measures is introduced by Artzner et al. [4]. A risk measures $\rho$ is coherent if it satisfies the four axioms translation invariance, sub-additivity, positive homogeneity, and monotonicity. The relevance of these properties is widely discussed by Artzner et al. [4]. Particularly, sub-additivity of a risk measure implies that the risk measure weakly decreases if risks are pooled. It also implies that there is no incentive for a company to split its risk into pieces and evaluate them separately.

The expected shortfall (ES) is given by

2 $$\begin{aligned} \textit{ES}_\alpha (X) = \frac{1}{1-\alpha } \int _{\alpha }^1 \textit{VaR}_\tau (X)d\tau , \end{aligned}$$

for all $\alpha \in [0,1)$, and all random variables *X* (a loss), whenever the integral converges. If the integral does not converge, ES equals infinity. An infinite value of a risk measure is useless for practical purposes. If the random variable *X* is continuously distributed, the ES may be even more intuitively expressed as conditional VaR or the tail conditional expectation:

3 $$\begin{aligned} \textit{ES}_\alpha (X) = E(X \mid X \ge \textit{VaR}_\alpha (X)). \end{aligned}$$

For numerical convenience, the expression in (3) is often used in historical simulations. For the same reason, we use (3) as definition of expected shortfall in this paper.

The use of expected shortfall gained popularity (see, e.g., [1, 20]). The main argument is based on the fact that ES considers the size of worst case events, whereas the VaR uses only a quantile. A quantile provides insight in the frequency of worst case events, where the ES considers both the frequency and size. For this reason, the interpretation of the ES is not straightforward. Other arguments in favor of the ES are based on stability and robustness (see, e.g., [33]). Daniélsson et al. [16] show that the sub-additivity may be violated if historical simulations are used. Historical simulations are used in the calibration of solvency II. Other authors that claim that the ES is more appropriate to determine capital buffers are Beder [10], Acerbi and Tasche 3, [44], Frey and McNeil [28], Yamai and Yoshiba [46], and Wagner [45]. CEIOPS [12] acknowledges the theoretical advantages of using the expected shortfall to calculate the SCR.

On the other hand, backtesting of the VaR is relatively straightforward compared to the ES (see, e.g., [2]). Kellner and Rösch [32] show that the ES is more sensitive to model risk than the VaR. Koch-Medina and Munari [34] show that the ES does not protect the policyholders sufficiently. Moreover, in the literature, the relevance of coherence (and sub-additivity) is criticized as well (see, e.g., [9, 35]). To summarize, there is no clear argument whether the ES is better than the VaR. This paper does not aim to provide a justification for either of the two risk measures, but seeks to compare them quantitatively in the solvency II context.

When returns are Gaussian, the same information is given by the value-at-risk and expected shortfall. In particular, the value-at-risk and expected shortfall are multiples of the standard deviation for zero-mean Gaussian random variables. For example, VaR at 99.5% confidence level is approximately 2.58 times the standard deviation, while expected shortfall at the same confidence level is approximately 2.89 times the standard deviation.^1^ The assumption that financial returns are Gaussian is often criticized and it may lead to an underestimation of the risk being faced (see, e.g., [40]). It is a well-known (e.g., [14]) that asset returns are fat-tailed, asymmetric and, therefore, not Gaussian.

## Solvency II

We describe the solvency II regulations in Sect. 3.1. In Sect. 3.2, we specify all formulas of the solvency capital requirements (SCR) according to EIOPA [25] for three relevant risk classes.

### Introduction

Solvency II is a regulatory framework that is in force since 2016 for the European insurance industry and puts demands on the required economic capital, risk management and reporting standards of insurance companies. The underlying quantitative regulation mechanism is that insurers should hold an amount of capital that enables them to absorb unexpected losses and meet the obligations towards policyholders at a high level of equitableness. In the European Union, there are on-going discussions about applying such a framework to all European pension funds as well [23]. For instance, see Doff [17], Eling et al. [19], Sandström [40], Steffen [43], and Wagner [45] for an overview and critical discussion of the solvency II framework.

This paper focusses on the first pillar of the solvency II framework. This pillar prescribes the quantitative requirements which an insurer must meet. It contains the SCR, which is to be calculated by using the standard formula or an internal model or a combination of the two. These quantitative requirements are supported by the so-called quantitative impact studies (QIS). For internal models, it is also not clear how the SCR is precisely defined [11] and therefore we focus on the standard formula in this paper.

EIOPA [25] describes how the assets and liabilities of an insurer should be valued. Assets should be valued at the amount for which they could be exchanged between knowledgeable willing parties in an arm’s length transaction. Liabilities should be valued at the amount for which they could be transferred, or settled, between parties in an arm’s length transaction. Valuing assets on a market-consistent basis is generally straightforward. Generally, perfect replication of expected cash flows is not possible for the liabilities of an insurer. The quantitative impact study 5 (QIS 5) prescribes to use the best estimate and the risk margin to value the liabilities. The best estimate of the liabilities (BEL) is the present value of the expected future liability payments. The risk margin is the cost of holding an amount of basic own funds equal to the SCR over the lifetime of the insurance liabilities.

EIOPA [25] prescribes that the SCR should correspond to the value-at-risk of the basic own funds of an insurer subject to a confidence level of 99.5% on a 1-year period. The principle of the standard formula is to apply a set of shocks to certain risk drivers, and to calculate the impact on the basic own funds for various risks. These shocks are calibrated using the VaR with a confidence level of 99.5%. The standard formula of the SCR is divided into risk modules. Predefined correlation matrices are used to aggregate the SCRs for all risks to the total SCR.

**Fig. 1:**
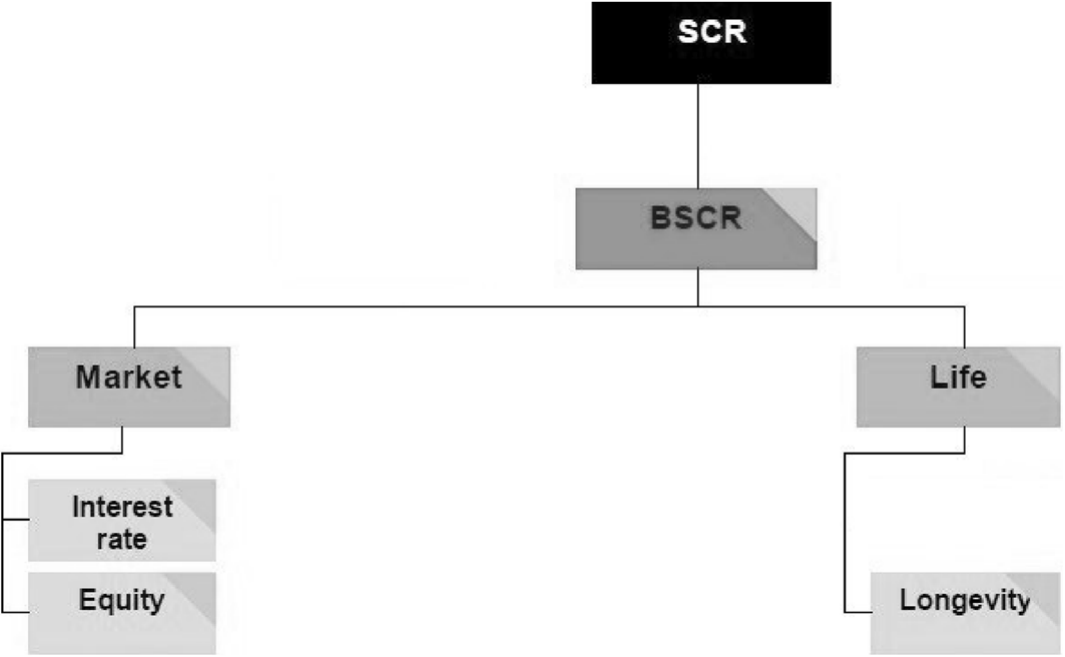
The different risk modules that we calibrate in this paper for the reduced total SCR

We do not consider operational risk, and the adjustment for the risk absorbing effects of technical provisions and deferred taxes. Moreover, we only consider equity risk, interest rate risk and longevity risk. In Fig. 1, we provide an overview of our reduced total SCR calculation and its different risk modules. Market risk and life risk account together for approximately 91.1% of the basic SCR for life insurance companies [22]. Moreover, EIOPA [24] shows that the market risk predominantly consists of equity and interest rate risk.

Market risk arises from the volatility of market prices of financial instruments. Exposure to market risk is measured by the impact of movements in the level of financial variables such as equity prices, interest rates, real estate prices and exchange rates. Market risk is the largest component of the SCR and market risk accounts for approximately 67.4% of the Basic SCR for representative life insurance companies [22].

Life risk covers the risk arising from the underwriting of life insurance, associated with both the perils covered and the processes followed in the conduct of the business. Life risk is the second largest risk class and it accounts for approximately 23.7% of the diversified BSCR for representative life insurance companies [22]. The most important components of life risk for a life annuity insurer are longevity risk and lapse risk. Longevity risk covers approximately 44% of the life SCR [22], and is associated with insurance obligations where an insurer guarantees to make recurring payments until the death of the policyholder and where a decrease in mortality rates leads to a decrease in the basic own funds. Longevity risk is associated to higher than expected pay-outs because of increasing life expectancy. In this paper, we ignore lapse risk for simplicity.

### Calculation of the SCR

In this section, we describe the solvency II regulations for calculating the reduced total SCR. The market SCR is a combination of the different market risks, in this case equity risk and interest rate risk. The market SCR, denoted by $SCR_{mkt}$, is defined as follows:

4 $$\begin{aligned} \textit{SCR}_{mkt}&= \sqrt{\textit{Mkt}_{eq}^2+ \textit{Mkt}_{int}+2A\cdot \textit{Mkt}_{eq} \cdot \textit{Mkt}_{int}}, \end{aligned}$$

where index *eq* denotes equity risk, index *int* denotes interest rate risk, $\textit{Mkt}_{i}$ is the solvency capital requirement for the individual market risk $i\in \{eq,int\}$, $A=0$ if the interest rate shocks are determined via the interest rate up scenario, and $A=0.5$ otherwise. We clarify the up and down shocks in interest rates later in (7)–(9). EIOPA [25] provides a standard model where the “global” equity shock is − 39% and the “other” equity shock is − 49%. There is a symmetric adjustment applied by EIOPA [25] that corrects for pro-cyclicality in equity returns (see [18]).

The basic own funds (BOF, net value of assets minus liabilities) is defined by CEIOPS [13] as the asset value minus the best estimate of the liabilities (BEL).^2^ The solvency capital requirement for equity risk is determined by the decrease of the *BOF* after a negative shock has been applied to equity. This negative shock implies that the value of equity decreases with a certain percentage. The shock differs for “global” equity and “other” equity. “Global” equity includes equities listed in EEA or OECD countries, and “other” equity includes equities listed in other countries, hedge funds, private equities and other alternative investments. For category $i\in \{``{\text{global}}", ``{\text{other}}"\}$, the solvency capital requirement is given by

5 $$\begin{aligned} \textit{Mkt}_{eq,i} = \max \{\triangle \textit{BOF } | \textit{equity \,shock}_{i} , 0\}, \end{aligned}$$

where $\triangle \textit{BOF}$ is the *BOF* before the equity shock minus the *BOF* after the equity shock. The equity SCR is given by

6 $$\begin{aligned} \textit{Mkt}_{eq}= \sqrt{\textit{Mkt}_{eq,``{\text{global}}"}^2+ \textit{Mkt}_{eq,``{\text{other}}"}^2+1.5\cdot \textit{Mkt}_{eq,``{\text{other}}"}\cdot \textit{Mkt}_{eq,``{\text {global}}"}}, \end{aligned}$$

where an implicit assumption is that the correlation between “global” and “other” equity is 0.75.

To determine the solvency capital requirement for interest rate risk, an upward and a downward shock are given to the interest term structure. The altered term structures are given by $\max \{(1 + \text {s}^{up}_m)\cdot r_m,r_m+1\%\}$ and $(1 + s^{down}_m)\cdot r_m$, where $r_m$ is the current interest rate with maturity *m*, and $s^{up}_m$ and $s^{down}_m$ are the up- and down-shocks to the interest rates with maturity *m*. These shocks to the interest rates are expressed as the relative amounts compared to the current interest rates. So, in addition to the calibration of the relative stress factor, a minimum shock of 1% is applied for the interest rate in the upward scenario [26]. Using an alternative term structure results in a change of the value of the assets and liabilities. The solvency capital requirements for the downward and upward shock are determined by the changes in the BOF if the shocked interest rate curve is used instead of the nominal term structure. The interest rate shock is the worst of the up and down shock. This leads to the following definitions:

7 $$\begin{aligned} \textit{Mkt}_{up,int}&= \triangle \textit{BOF } | \textit{up shock},\end{aligned}$$

8 $$\begin{aligned} \textit{Mkt}_{down,int}&= \triangle \textit{BOF } | \textit{down shock},\end{aligned}$$

9 $$\begin{aligned} \textit{Mkt}_{int}&=\max \{\textit{Mkt}_{up,int}, \textit{Mkt}_{down,int},0\}. \end{aligned}$$

In this paper, we only consider longevity risk in the class of life risk, i.e., the life SCR equals longevity SCR. The longevity SCR is determined by the change in net value of assets minus liabilities after a permanent percentage decrease in mortality rates for all ages. This decrease resembles the risk that people live longer than expected and this leads to an increase in the present value of the liabilities from annuity products. This shock therefore leads to a decrease in the value of *BOF*. The definition of longevity SCR is given by

10 $$\begin{aligned} SCR_{life}=\textit{Life}_{long} = \max \{\triangle \textit{BOF } | \textit{longevity shock},0\}. \end{aligned}$$

EIOPA [25] provides a standard model where all mortality rates are reduced by $20\%$.

The market risk and life risk SCRs are combined to determine the reduced total SCR. The reduced total SCR is calculated by the following formula:

11 $$\begin{aligned} \textit{SCR}= \sqrt{\textit{SCR}_{mkt}^2+ \textit{SCR}_{life}^2+0.5\cdot \textit{SCR}_{mkt} \cdot \textit{SCR}_{life}}, \end{aligned}$$

where $\textit{SCR}_{i}$ is the solvency capital requirement for the individual risk class $i\in \{mkt,life\}$, and where an implicit assumption is that the correlation between market risk and life risk is 0.25.

The structure of the solvency capital requirements in solvency II is partially derived from a formula developed by the German Insurance Association (see, e.g., [19, 41]). The structure of a square-root formula as in (4), (6) and (11) is derived from the assumption that the individual returns are Gaussian and the dependence is linear. In general, linear dependence is sufficient to describe dependencies between elliptical distributions. However, there arise problems with this formula if the individual returns are not Gaussian, or dependencies are non-linear. For instance, skewness or excess kurtosis of the marginal distributions may lead to very irregular outcomes (see [40]). Moreover, even if the different returns have Gaussian marginal distributions, their influence on the aggregate loss may not be closely related to a square-root formula (see [7]). Non-linear dependence structures may yield situations where the square-root formula severely underestimates the total risk (see [38]). A more accurate approach instead of the square-root formula may require nested simulations, and is therefore substantially more complex (see [8]). In this paper, we do not analyze alternative risk aggregation methods.

## Methodology

In this section, we calibrate the SCR stress scenarios for the three risk classes based on the VaR and the ES following the calibration methods used in solvency II. We calculate the SCR with stress scenarios calibrated with VaR or ES for a fictitious life annuity insurer. To show the impact of the stress scenarios on this life annuity insurer, we first specify the insurer in Sect. 4.1. In Sect. 4.2, we introduce the method we use to compare the stress scenarios based on VaR with the stress scenarios based on ES. In Sect. 4.3, we show the stress scenarios for all three risk classes.

### Calibrating the fictitious life annuity insurer

The liability portfolio of the fictitious insurer consists of (deferred) life annuity products only. The portfolio is normalized such that it consists of 100,000 male policyholders. The policyholders in this portfolio have an average age of 50 years. In Table 7 in Appendix 1, we provide details of the liability portfolio.

The single-life annuity will be paid at the end of every year starting from age 65 if alive. So, for policyholders younger than age 65, it is a deferred annuity. For the youngest age cohort 21, the yearly amount paid out after age 65 is equal to 0.067. This amount increases linearly over the age cohorts up to 1 for age cohorts of 65 years old and older. The increasing trend accounts for the fact that younger cohorts have paid less premiums in the past. Here, we use age 65 as a retirement age, where the retirement benefits are normalized to 1 for every retired individual. We refer to Hári et al. [29] for an overview of the actuarial valuation of the annuity liabilities.

To discount cash flows and determine the bond prices, we use the nominal interest rate term structure of the European central bank and a Smith–Wilson extrapolation [42] towards an ultimate forward rate (UFR) for interest rates after 20 years (see Fig. 7 in Appendix 1).^3^ We set the present at December 31st, 2014.

We assume that the fictitious insurer has no BOF, so the asset value is equal to the value of the liabilities. The asset portfolio consists of 25% in “global” equity and 75% in bonds. To construct the bond portfolio, we pick 5- and 30-year bonds.^4^ The 5-year bond has 2.13% coupons, and the 30-year bond has 3.45% coupons.^5^ We determine the bond portfolio as follows. The duration of the bonds equals 50% of the duration of the liabilities. Since 75% of the assets is invested in bonds, this coincides with a duration of the assets of $75\% \times 50\%=37.5\%$ of the duration of the liabilities. Via this duration, we determine the amount invested in the 5- and 30-year bonds. For both the characteristics of the portfolio and the composition of the asset portfolio, we will later perform a sensitivity analysis. We use the mortality table of the Dutch Actuarial Society: AG 2012-2062,^6^ developed in 2012, which contains a longevity trend. Note that there is no risk involved in equity, interest rates and longevity other then the stress scenarios.

### Matching the total SCR value

For the fictitious life annuity insurer, we denote the SCR based on stress scenarios calibrated on VaR and ES by SCR($\textit{VaR}_{\alpha }$) and SCR($\textit{ES}_{\theta }$), for different parameters $\alpha$ and $\theta$. We focus on the relative impact on the SCR for the three risk classes. Therefore, to compare the SCR($\textit{VaR}_{\alpha }$) properly with the SCR($\textit{ES}_{\theta }$), the confidence level for the ES, $\theta$, is chosen based on matching the reduced total SCR value, i.e.,

12 $$\begin{aligned} \textit{SCR}(\textit{VaR}_{\alpha }) = \textit{SCR} (\textit{ES}_{\theta }). \end{aligned}$$

**Fig. 2:**
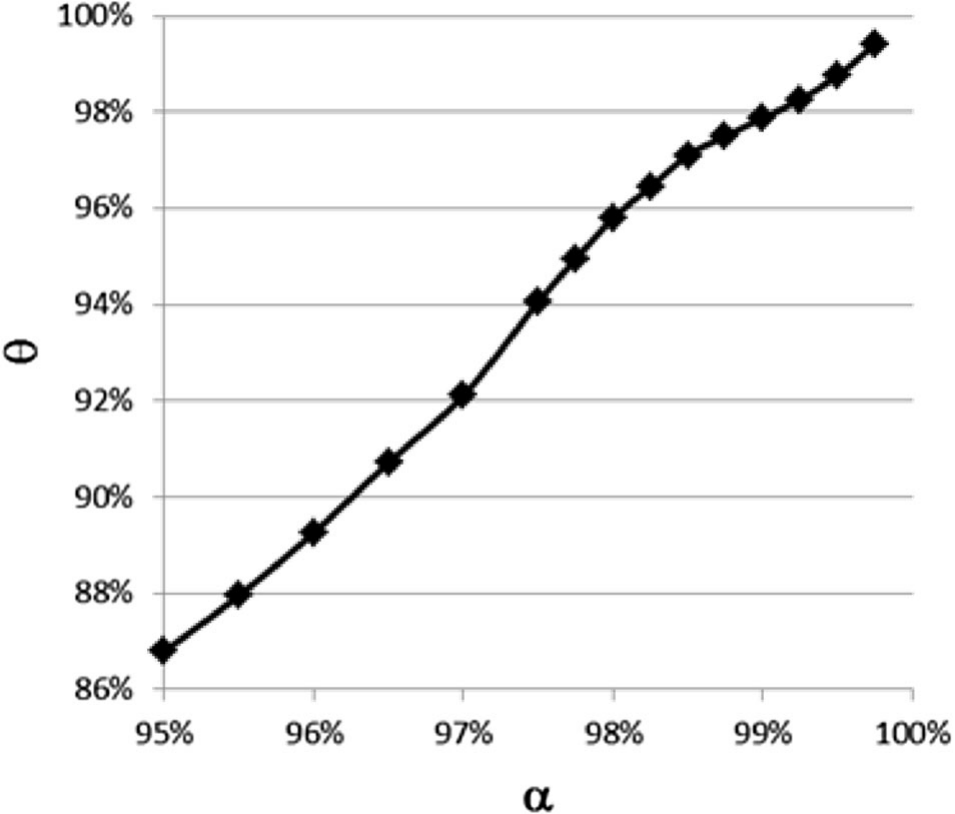
The function $\theta$ as defined in Sect. 4.2

In this way, we define the function $\theta :[95\%,100\%]\rightarrow [0,1]$ as a strictly increasing function such that (12) is satisfied for all $\alpha \in [95\%,100\%]$. For instance, we find that $\theta (99.5\%)\approx 98.78\%$ and $\theta (98.5\%)\approx 97.00\%$ if we calibrate the SCR risk scenarios as we will specify in Sect. 4.3. We display the function $\theta$ in Fig. 2. We get that the function $\theta$ is not affine, which is due to non-Gaussianity of the returns.^7^

Using the function $\theta$ in (12), we compare the stress scenarios for equity risk, interest rate risk and longevity risk. Those stress scenarios lead to three SCRs, which are then used to determine the reduced total SCR as described in Sect. 3.2. We denote the vector of the SCRs for all three risk classes as an allocation.

### Calibrating the SCR stress scenarios

In this section, we calibrate stress scenarios for equity risk, interest rate risk, and longevity risk based on the value-at-risk and the expected shortfall. After we calibrate the distribution of the risks, we derive stress scenarios based on the VaR and ES. These stress scenarios will be applied to the representative life annuity insurer in Sect. 5. EIOPA [26] proposes stress scenarios given by a shock of the underlying risk. This risk may be multi-dimensional, which is the case for interest rate risk. Interest rate SCR is calculated via shocks for every duration (see (7)–(9)). In line with this approach of EIOPA, we assume that the SCR for a risk is given by

$$\begin{aligned} \textit{shock}_i&= \rho (\textit{risk}_i),i=1,\ldots ,I,\\ SCR&= \Delta\textit{BOF}|\textit{shock}, \end{aligned}$$

where $\rho$ is the risk measure VaR or ES, and the dimension of the risk is *I*. The data we use in this paper to calibrate the distribution of the risks is similar to the data used of CEIOPS [13] and EIOPA [26], except that we modify the horizons.

The calibration method for equity risk is discussed by CEIOPS [13]. The empirical distribution of annual holding period returns is derived from the Morgan Stanley Capital International (MSCI) World Developed (Market) Price Equity Index. The data from Bloomberg consists of daily returns for a period of 41 years, starting in June 1973 until June 2014. We convert this into annual holding period returns for every working day, with an overlapping horizon. The empirical VaR and the empirical ES serve as the stress shock for “global” equity. We follow the technical specifications of EIOPA [25] with an adjustment of 7.5% to increase the equity risk shock. With this symmetric adjustment included, the stress rates for equity risk are provided in Table 1. Recall that the function $\theta (\alpha )$ is fixed such that the reduced total SCR remains the same. If $\alpha =99.5\%$, the differences between using VaR and ES are small. When the quantile $\alpha =98.5\%$ is chosen, the use of VaR leads to a larger equity risk shock.

**Table 1:** Stress scenarios for “global” equity risk derived from the VaR and ES, where a fixed symmetric adjustment of 7.5% is included

$\alpha$ (%)	$\textit{VaR}_{\alpha }$ (%)	$\textit{ES}_{\theta (\alpha )}$ (%)
99.5	− 51.66	− 51.69
98.5	− 46.75	− 45.22

For the calibration method for interest rate risk, we use the following four datasets as also used by CEIOPS [13] and EIOPA [26], but with shifted horizons in order to include more recent data:

- Euro area government bond yield curve, with maturities from 1 to 15 years, spaced out in annual intervals. The daily data spans a period of approximately 10 years and runs from September 2004 to July 2014. The data is from the European Central Bank;^8^
- the UK government liability curve. The data is daily and is from the Bank of England.^9^ The data covers a period from January 1998 to June 2014, so that the longer maturities (i.e., beyond 15 years) are all available. It contains rates of maturities starting from 1 year up until 20 year whilst the in between data points are spaced on annual intervals;
- Euro vs Euribor IR swap rates. The daily data is downloaded from Datastream and covers a period from 1999 to 2014. The data contains the 1–10 year rates spaced out in 1-year intervals, as well as the 12, 15 and 20 year rates;
- UK (GBP) 6m IRS swap rates. The daily data is downloaded from Datastream and covers a period from 1999 to 2014. The data contains the 1–10 year rates spaced out in 1-year intervals, as well as the 12, 15 and 20 year rates.

The data sets represent the most liquid markets for interest rate-sensitive instruments in the European area. These four datasets are used to determine the risk in interest rates, that we will use to determine the stress scenarios.

We calibrate the stress interest rate scenarios using principal component analysis (PCA) as prescribed by EIOPA [26]. To transform the principal components and eigenvectors into a VaR and an ES, we use the method described by Fiori and Iannotti [27]. We describe and discuss the method we use in Appendix 2. For every dataset, we derive a shock scenario for the annualized interest rate returns. So, we have four stress shocks for every maturity from 1 to 20 years. For each maturity, the overall interest rate shock is the average of the four stress shocks.

In Table 2, we provide the resulting stress scenarios of interest rates with quantiles $\alpha =98.5, 99.5\%$ for various maturities. Similar as for equity risk, the differences between using VaR and ES are small when $\alpha =99.5\%$. Even though the differences are small, calibrating with VaR leads to larger stress rates. When the quantile $\alpha =98.5\%$ is chosen, the differences are larger. We get for the fictitious insurer that the down-shock is more harmful than the up-shock. This is partially due to the fact that duration is not fully hedged.^10^ Hence, the down-shock is generally used for the interest rate SCR.

**Table 2:** Interest rate stress scenarios $s^{up}_m$ and $s^{down}_m$ for maturities $m=1,\ldots ,20$, calibrated with $\textit{VaR}_{99.5\% }$, $\textit{ES}_{\theta (99.5\%)}$, $\textit{VaR}_{98.5\%}$, or $\textit{ES}_{\theta (98.5\%)}$

Maturity	*SCR*($\textit{VaR}_{99.5\%}$)		*SCR*($\textit{ES}_{\theta (99.5\%)}$)		*SCR*($\textit{VaR}_{98.5\%}$)		*SCR*($\textit{ES}_{\theta (98.5\%)}$)	
	Up (%)	Down (%)	Up (%)	Down (%)	Up (%)	Down (%)	Up (%)	Down (%)
1	49.1	− 83.0	49.5	− 82.6	42.0	− 74.7	43.8	− 77.7
2	51.0	− 84.5	51.1	− 84.9	43.3	− 76.4	45.1	− 78.7
3	47.3	− 75.6	47.4	− 75.5	40.4	− 65.6	42.0	− 67.9
4	44.2	− 66.3	44.3	− 66.6	37.6	− 59.1	39.1	− 60.7
5	41.3	− 59.8	41.2	− 60.1	35.0	− 54.0	36.5	− 55.2
6	39.6	− 55.1	39.6	− 55.3	33.6	− 49.8	35.0	− 51.1
7	37.6	− 51.1	37.6	− 51.2	32.5	− 46.7	33.5	− 47.6
8	35.3	− 48.5	35.2	− 48.5	30.4	− 43.6	31.4	− 44.8
9	33.8	− 47.2	33.6	− 46.9	28.9	− 42.3	30.0	− 43.2
10	32.4	− 44.9	32.6	− 45.2	27.9	− 40.8	29.0	− 41.6
11	32.8	− 44.0	32.7	− 44.5	27.9	− 40.0	29.1	− 40.9
12	32.7	− 43.6	32.7	− 43.8	28.0	− 39.6	29.1	− 40.3
13	32.6	− 42.6	32.6	− 42.7	27.8	− 38.5	28.8	− 39.2
14	32.2	− 41.3	32.4	− 41.6	27.5	− 37.3	28.6	− 38.2
15	31.8	− 40.3	32.2	− 40.5	27.1	− 36.3	28.2	− 37.2
16	29.5	− 39.2	29.7	− 39.4	24.9	− 35.7	25.9	− 36.4
17	29.6	− 38.9	29.9	− 38.8	24.9	− 35.1	25.9	− 35.8
18	29.8	− 38.5	30.0	− 38.3	24.9	− 34.5	26.0	− 35.3
19	30.0	− 37.8	30.2	− 37.8	24.9	− 34.0	26.1	− 34.7
20	30.5	− 37.4	30.4	− 37.3	25.5	− 33.4	26.2	− 34.2

The stress scenarios are then applied to the assets and liabilities of the fictitious life annuity insurer as described in Sect. 3.2

We follow the calibration method for longevity shocks as introduced by CEIOPS [13]. From the Human Mortality Database, we use unisex mortality tables from 1992 until 2009 with age bands of 5 years from nine countries. These nine countries are Denmark, France, England and Wales, Estonia, Italy, Sweden, Poland, Hungary, and Czech Republic. We calibrate the longevity stress scenarios by assuming that annual mortality rate improvements follow a Gaussian distribution as prescribed by CEIOPS [13]. The same shock in mortality rates is used for all different ages.

Annual mortality rate changes are calculated per country, per age band and per year based on the data from the Human Mortality Database. We compute the means and standard deviations of the annual mortality rate improvements, and we assume that all annual mortality rate improvements have a Gaussian distribution. In this case we have 198 Gaussian distributions, since we have nine countries and 22 different age bands. CEIOPS [13] determines per age cohort an average longevity shock over all nine countries. This is than summarized into a longevity shock for all age cohorts. We propose to determine the average of all age cohorts to determine the age-independent longevity shock. So, we take the average of all VaRs or ESs. Due to the Gaussian distribution of mortality improvements, we could define $\hat{\theta }$ analytically for every $\alpha$ such that $\text {SCR}_{life}(\textit{VaR}_{\alpha })=\text {SCR}_{life}(\textit{ES}_{\hat{\theta }})$ for any insurance portfolio. Differences in the longevity SCR are due to the function $\theta$ which may differ from $\hat{\theta }$.

We display the stress scenarios for longevity risk in Table 3. For both quantiles $\alpha =98.5, 99.5\%$, calibrating with ES leads to fiercer shock rates. The effect is larger when the quantile $\alpha =98.5\%$ is used.

**Table 3:** Stress rates for longevity risk derived from the VaR and ES

$\alpha$ (%)	$\textit{VaR}_{\alpha }$ (%)	$\textit{ES}_{\theta (\alpha )}$ (%)
99.5	− 18.78	− 18.90
98.5	− 16.19	− 16.90

**Fig. 3:**
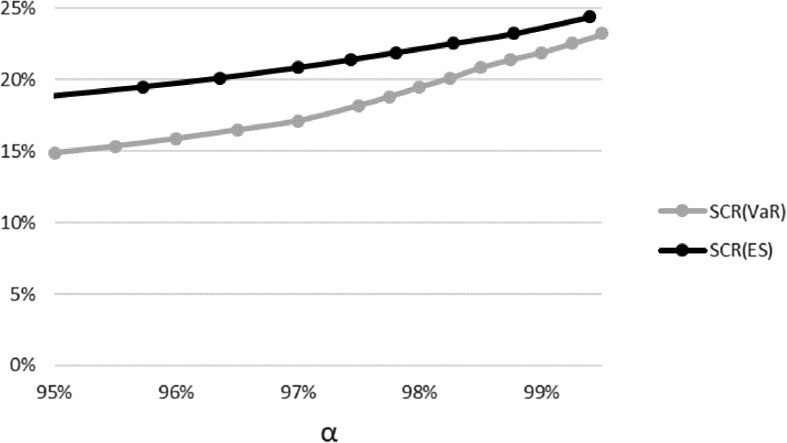
Comparing SCR($\textit{VaR}_{\alpha }$) with SCR($\textit{ES}_{\alpha }$) of the fictitious life annuity insurer for various $\alpha$

When we apply the stress scenarios, we obtain that the reduced total SCR of the fictitious life annuity insurer is approximately $23.24\%$ of the BEL. The allocation of this SCR($\textit{VaR}_{99.5\%}$) to the three different risk classes in shown in Table 4. In Fig. 3 we display SCR($\textit{VaR}_{\alpha }$) and SCR($\textit{ES}_{\alpha }$). By construction, it holds that $\text {SCR}(\textit{VaR}_{\alpha })\le \text {SCR}(\textit{ES}_{\alpha })$.

**Table 4:** The allocation of this SCR($\textit{VaR}_{99.5\%}$) for the fictitious life annuity insurer

Reduced total SCR	23.24%
Interest rate SCR	10.05%
Equity SCR	14.85%
Market SCR	21.70%
Longevity SCR	4.49%

All SCRs are expressed in percentage of the BEL

## Results

In Sect. 5.1, we compare the allocation of SCR($\textit{VaR}_{\alpha }$) with the allocation of SCR($\textit{ES}_{\theta (\alpha )}$ for a fictitious life annuity insurer. In Sect. 5.2, we provide some sensitivity analysis and show that the results of Sect. 5.1 are robust to changes to the asset and liability portfolio of the fictitious life annuity insurer.

### Comparing the SCR($\textit{VaR}_{\alpha }$) with the SCR($\textit{ES}_{\theta (\alpha )}$) for the fictitious life annuity insurer

In this section, we compare the SCR($\textit{VaR}_{\alpha }$) with the SCR($\textit{ES}_{\theta (\alpha )}$) for the fictitious life annuity insurer. Recall that $\theta$ is chosen such that SCR($\textit{VaR}_{\alpha }$) equals SCR($\textit{ES}_{\theta }$). Therefore, we focus on the allocation of the reduced total SCR for the three risk classes: equity SCR, interest rate SCR and longevity SCR. By comparing these allocations for VaR and ES, we can see whether the current method, which uses VaR, underestimates or overestimates certain risks. This is done for different quantiles $\alpha$ used for the VaR.

#### The changes in allocation of the reduced total SCR

**Fig. 4:**
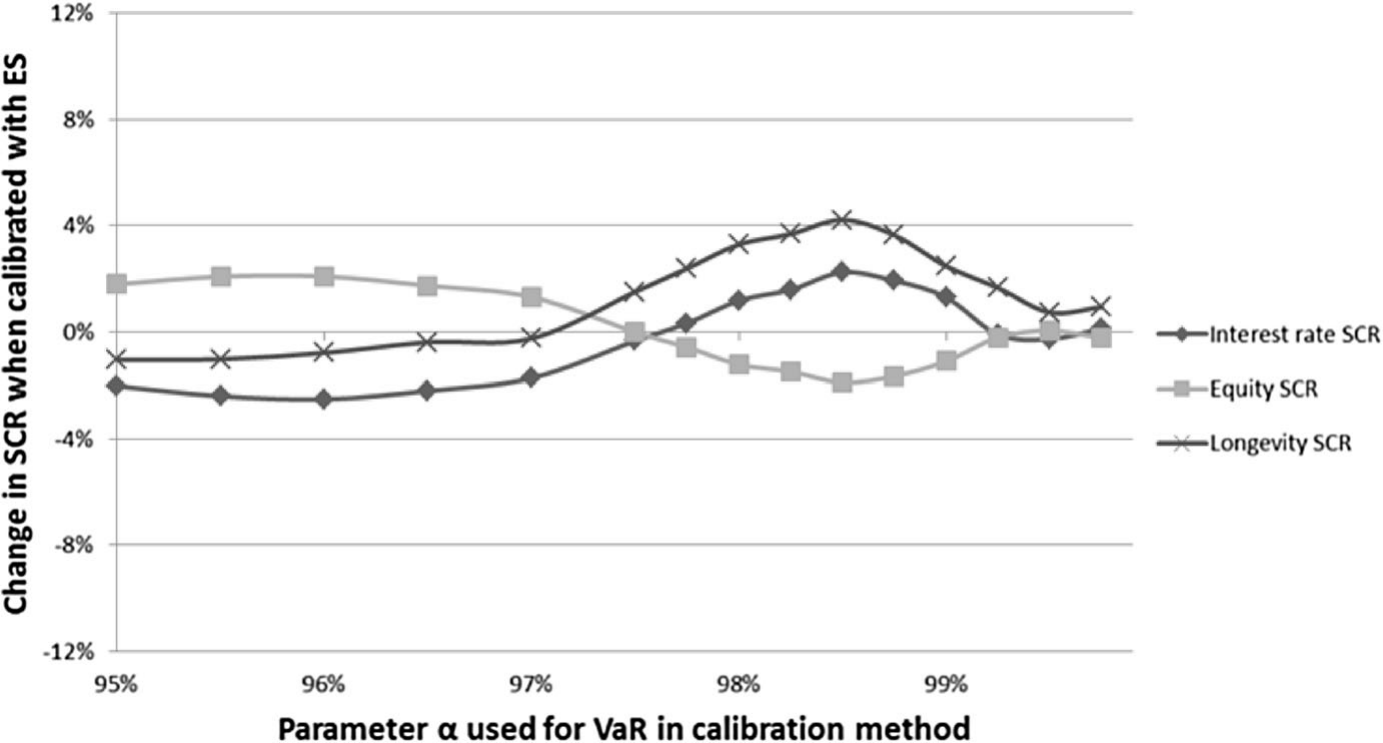
Comparing the allocation of SCR($\textit{VaR}_{\alpha }$) with the allocation of SCR($\textit{ES}_{\theta (\alpha )}$) for the fictitious life annuity insurer, when the stress scenarios are determined in a similar way as EIOPA prescribes (base case). The horizontal axis represents the quantile $\alpha$ used for VaR in the calibration method. The vertical axis represents the change in allocation of the SCR when the stress scenarios are calibrated with ES instead of VaR

Figure 4 shows the change in allocation of the reduced total SCR for the three different risk classes when ES is used to calibrate the shock scenarios instead of VaR. The stress scenarios used to determine the SCR are derived as prescribed by EIOPA, that means that the interest rate stress scenarios are calibrated using PCA and the longevity stress scenarios are calibrated assuming Gaussian distributions. We call this the base case.

Figure 4 shows for the quantile $\alpha =99.5\%$ that the differences in allocation are small. When the quantile $\alpha$ decreases to 98.5%, the differences become more significant. By using ES to determine the shocks, the longevity risk and the interest rate shocks are more harmful and the equity risk shock is milder. When we use ES instead of VaR to determine the stress shocks for $\alpha =98.5\%$, the longevity SCR grows with 4.22%, the interest rate SCR increases with 2.27%, and the equity SCR decreases with 1.77%.

These changes do not add up to zero since the amounts of SCR for the different risk measures differ. Therefore, a decrease of 2% for the equity SCR leads to a larger change in the actual amount of SCR than an increase of 2% for the interest rate SCR, because the equity SCR is larger than the interest rate SCR. When the quantile $\alpha$ is smaller than 98.5%, the differences are smaller. For values of $\alpha$ smaller than 97.5%, we get that the equity SCR is relatively large. This follows from the observation that the tails for equity returns are fat (see, e.g., [14]), but the extreme events are worse for the interest rate shocks. We get that relative effects are fairly constant when $\alpha$ gets smaller, as tail risk events will have a smaller impact on both the ES as the VaR.

#### The changes in allocation of the reduced total SCR with empirical stress scenarios

Historical simulation is a popular method in practice. The empirical performance of historical simulation has been examined by, e.g., Beder [10], Hendricks [30], and Pritsker [39]. Historical simulation is a resampling method which does not assume any distribution about the underlying risk process. For this non-parametric model, we assume that the distribution of past returns is a perfect representation of the expected future returns. The advantage is that we do not impose any assumptions about the underlying probability distributions. Historical simulation is heavily sensitive to the length of the sample of past returns (see, e.g., [39]).

If we apply historical simulation to the three risk classes, the equity SCR remains unchanged as the equity shock was already determined empirically. For the interest rate SCR, we use the annualized absolute interest rate changes of the four datasets as described in Sect. 4.3. Per dataset and for each maturity, we calculate the empirical VaR and ES and this leads to a vector of absolute up and down shocks. The percentage interest rate stress vectors per dataset are then computed by dividing it by the average interest rates for each maturity. Since the swap rates are not defined for all maturities between 1 and 20 years, linear interpolation is used to determine the swap rates for these maturities. The average of the four up and down shock vectors has been taken to determine the overall up and down shock vector. For the longevity SCR, we aggregate all annual mortality rate changes for the nine countries used by EIOPA (see Sect. 4.3) and all age cohorts for the period 1992–2009. For this dataset, we calculate an empirical VaR and an empirical ES. The empirical VaR or the empirical ES serve as the stress rate for longevity risk for all age cohorts.

The SCR($\textit{VaR}_{99.5\%}$) increases from approximately $23.24\%$ of the BEL under the EIOPA standard model to approximately $24.40\%$ if the stress scenarios for the three risk classes are determined empirically. We show the differences of the SCR of all classes of risk in Table 5. From this table we get that the longevity shocks are more harmful if the SCR is determined empirically. This effect dominates the increase in the reduced total SCR. The interest rate SCR decreases by approximately $13.09\%$ if it is determined empirically.

**Table 5:** Overview of the SCR changes of different risk classes using $\textit{VaR}_{99.5\%}$, if we switch from PCA analysis for interest shocks and the Gaussian approximations for longevity shocks to empirical interest and longevity shocks

Change reduced total SCR	$5.01\%$
Change interest rate SCR	$-\,13.09\%$
Change equity SCR	$0.00\%$
Change market SCR	$-\,4.82\%$
Change longevity SCR	$96.11\%$

All changes in SCR are expressed as percentage of the BEL

**Fig. 5:**
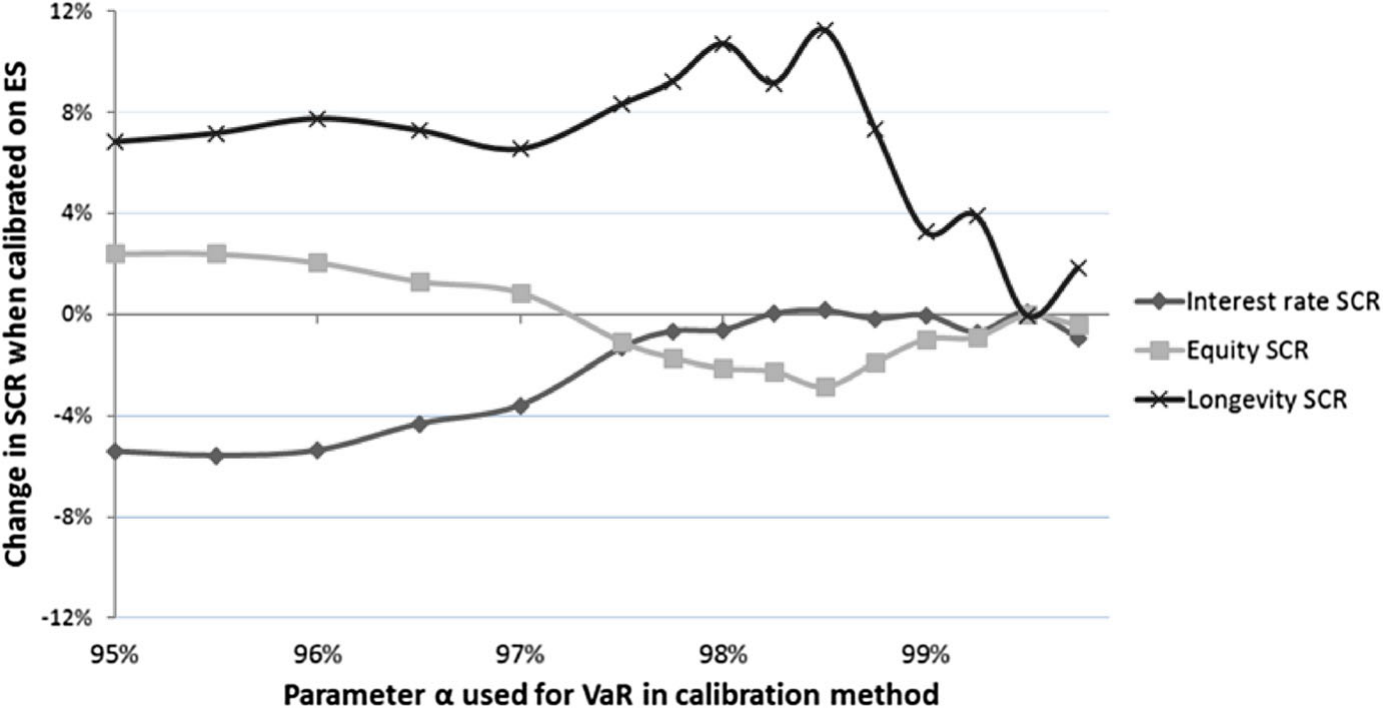
Comparing the allocation of SCR($\textit{VaR}_{\alpha }$) with the allocation of SCR($\textit{ES}_{\theta (\alpha )}$) for the fictitious life annuity insurer, when the stress scenarios are determined empirically. The horizontal axis represents the quantile $\alpha$ used for VaR in the calibration method. The vertical axis represents the change in allocation of the SCRs when the stress scenarios are calibrated with ES instead of VaR

Figure 5 shows the change in allocation of the reduced total SCR for the three different risk classes when ES is used to calibrate the shock scenarios instead of VaR. The stress scenarios used to determine the SCR are this time determined empirically. Similar as when EIOPA stress scenarios are used, the difference in individual risk modules is small when $\alpha$ = 99.5%. Again, the differences are largest when $\alpha$ is approximately 98.5%. At the quantile $\alpha =98.5\%$, the differences are considerably larger compared to the base case, since the longevity SCR is approximately $11.26\%$ higher and the equity SCR is approximately $2.85\%$ lower for SCR($\textit{ES}_{\theta (\alpha )}$) when compared with SCR($\textit{VaR}_{\alpha }$). For smaller values of the quantile $\alpha$ until approximately 97.5% we see that the differences are getting smaller. The changes in interest rate SCR differ substantially when empirical stress scenarios are used compared to when EIOPA stress scenarios are used. For values of the quantile $\alpha$ smaller than 97.5%, we see that the differences are stable, and differences in the interest rate SCR are smallest. We also see this in Fig. 4 in Sect. 5.1.1, where EIOPA stress scenarios are used.

Comparing Figs. 4 and 5, we get that the differences in the SCR allocation when ES is used instead of VaR are considerably larger when the stress scenarios are calibrated empirically. The longevity SCR will increase more when ES is used instead of VaR and when the quantile $\alpha$ is below 99%. This increase is at the expense of a lower interest rate SCR.

For both using EIOPA stress scenarios and empirical stress scenarios we see that the changes in allocation of the reduced total SCR are small for the quantile $\alpha =99.5\%$ as used in solvency II. When the quantile $\alpha$ decreases, the differences between the SCR allocation when ES is used instead of VaR get larger. We get that by using ES to determine the shocks, longevity risk is more harmful and equity risk is milder, particularly at the quantile $\alpha =98.5\%$. When we decrease the quantile $\alpha$ until approximately 97.5%, these effects are getting smaller.

**Fig. 6:**
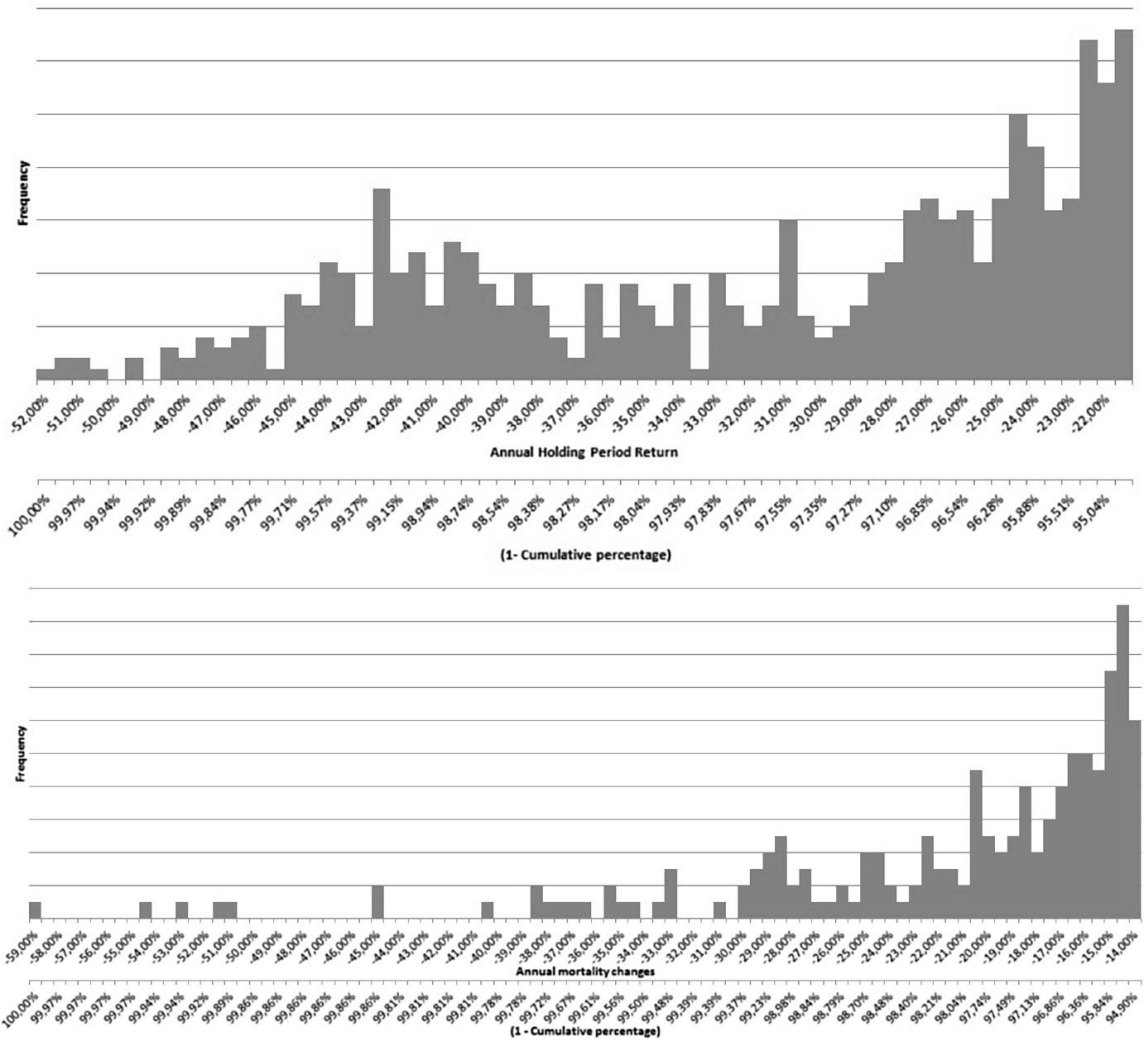
The left tail (smallest 5%) of the empirical distribution for the data used in this paper for the equity returns (upper graph) and the mortality rate changes (lower graph). The first horizontal axis represents the annual holding period return of equity (upper graph) or the annual longevity changes (lower graph). For the second horizontal axis, we display the survival distribution (1—cumulative distribution) of the empirical distribution. This helps us to determine the quantiles of the distribution. The vertical axes represent the frequency

**Fig. 7:**
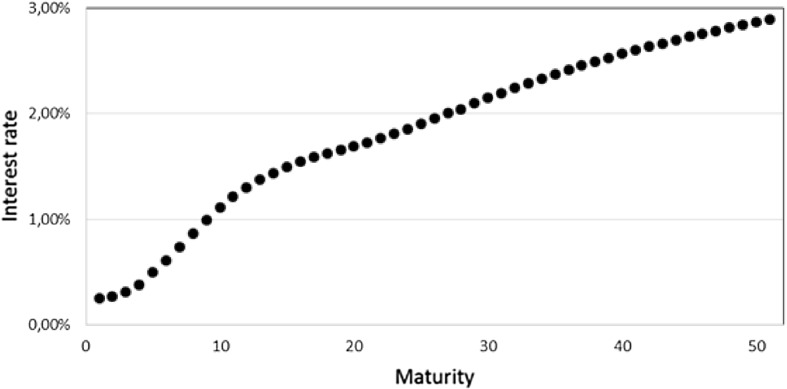
The nominal interest rate term structure from the European Central Bank of December 31st, 2014, used in Sect. 4.3. Source: http://www.dnb.nl/statistiek/statistieken-dnb/index.jsp (last accessed: May 4th, 2017)

By comparing the empirical distribution of the tail of the data used for equity risk and longevity risk we can explain why the differences are largest at approximately 98.5%. The downside tails of the data used for equity risk and longevity risk are shown in Fig. 6. In this figure, both graphs have two horizontal axis. The first horizontal axis represents the annual holding period return for equity risk and the annual mortality rate changes for longevity risk. On the second horizontal axis, we display the survival distribution (1—cumulative distribution).

One of the main characteristics of the VaR is that it does not consider the shape of the tail. When the distribution has a heavy tail beyond the VaR level, then this might lead to an underestimation of the risk. In Fig. 6, we see that the distribution of longevity has a heavy tail and that the distribution of equity returns has a less heavy tail. This explains why the longevity SCR is smaller and the equity SCR is larger if we use VaR. To understand why the difference is largest for $\alpha \approx 98.5\%$, we focus on the empirical survival distribution. For equity risk the $\textit{VaR}_{98.5\%}$ is situated in the middle of the heavy tail, therefore the equity SCR is relatively big when VaR is used. For longevity risk, however, the $\textit{VaR}_{98.5\%}$ is situated just before the start of the heavy tail, and this leads to a much smaller longevity SCR when VaR is used.

### Sensitivity analysis

In this section, we study the sensitivity of our results. We do this by changing the composition of the asset portfolio and the liability portfolio of the fictitious life annuity insurer.

#### Changing the asset portfolio

As described in Sect. 4.1, the fictitious life annuity insurer has an asset portfolio which consists of 25% in equity, and the remainder is invested in bonds. To test if our results are sensitive to the composition of the asset portfolio we vary with the percentage of equity in the portfolio and compare the allocation of SCR($\textit{VaR}_{\alpha }$) with the allocation of SCR($\textit{ES}_{\theta (\alpha )}$).

We analyze the following asset portfolios:

- 100% equity;
- 50% equity and 50% bonds;
- 25% equity and 75% bonds (base case);
- 10% equity and 90% bonds;
- 100% bonds.

When the asset portfolio contains bonds, the amounts invested in the 5- and 30-year bonds are determined by matching the duration of the bond portfolio with 50% of the duration of the liabilities. Hence, the duration is matched better when the insurer invests more in bonds than in equity.

**Fig. 9:**
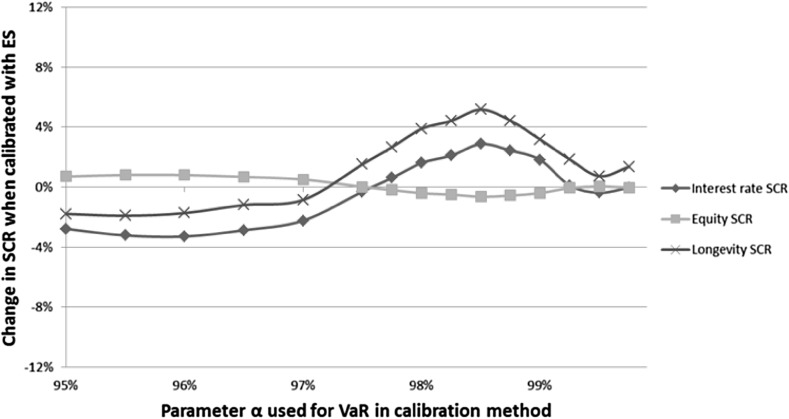
Comparing the allocation of SCR($\textit{VaR}_{\alpha }$) with the allocation of SCR($\textit{ES}_{\theta (\alpha )}$) for the fictitious life annuity insurer, with an asset portfolio consisting of 100% equity. The horizontal axis represents the quantile $\alpha$ used for VaR in the calibration method. The vertical axis represents the change in allocation of the SCRs when the stress scenarios are calibrated with ES instead of VaR

**Fig. 10:**
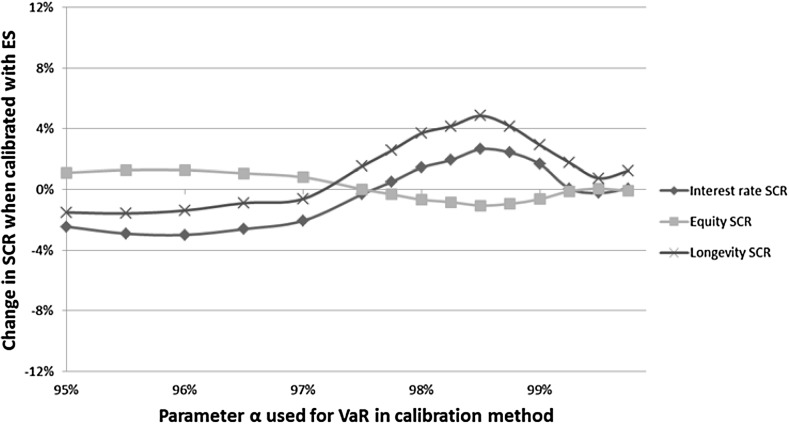
Comparing the allocation of SCR($\textit{VaR}_{\alpha }$) with the allocation of SCR($\textit{ES}_{\theta (\alpha )}$) for the fictitious life annuity insurer, with an asset portfolio consisting of 50% equity and 50% bonds. The horizontal axis represents the quantile $\alpha$ used for VaR in the calibration method. The vertical axis represents the change in allocation of the SCRs when the stress scenarios are calibrated on ES instead with VaR

**Fig. 11:**
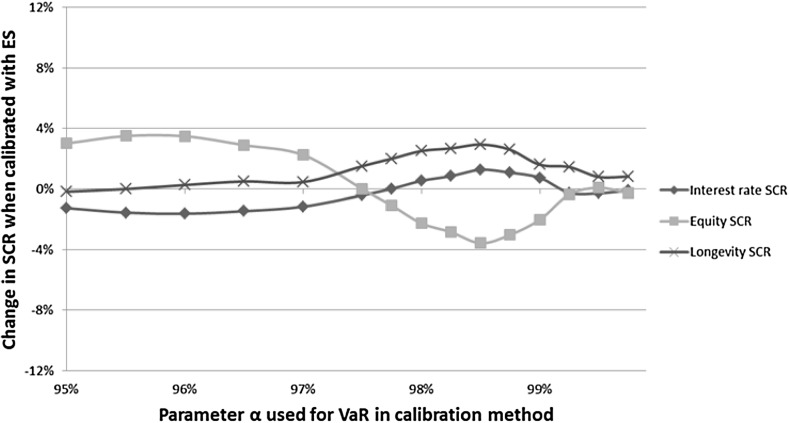
Comparing the allocation of SCR($\textit{VaR}_{\alpha }$) with the allocation of SCR($\textit{ES}_{\theta (\alpha )}$) for the fictitious life annuity insurer, with an asset portfolio consisting of 10% equity and 90% bonds. The horizontal axis represents the quantile $\alpha$ used for VaR in the calibration method. The vertical axis represents the change in allocation of the SCRs when the stress scenarios are calibrated with ES instead of VaR

**Fig. 12:**
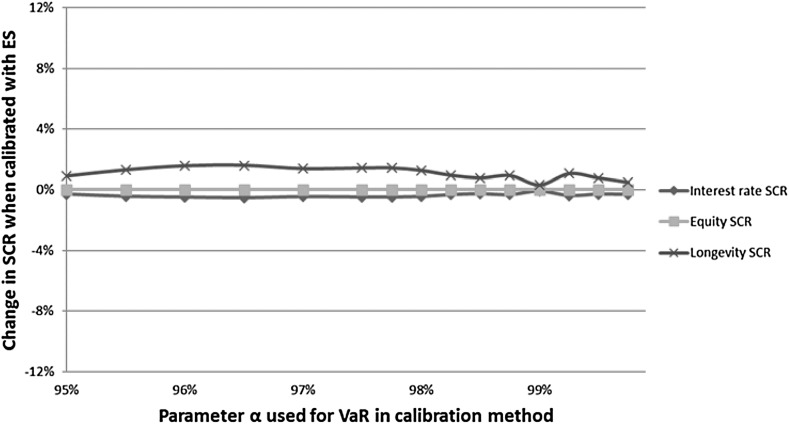
Comparing the allocation of SCR($\textit{VaR}_{\alpha }$) with the allocation of SCR($\textit{ES}_{\theta (\alpha )}$) for the fictitious life annuity insurer, with an asset portfolio consisting of 100% bonds. The horizontal axis represents the quantile $\alpha$ used for VaR in the calibration method. The vertical axis represents the change in allocation of the SCRs when the stress scenarios are calibrated with ES instead of VaR

Figures 9, 10, 11 and 12 in Appendix 3 show the change in allocation of the reduced total SCR for the three different risk classes when ES is used to calibrate the shock scenarios instead of VaR for the different asset portfolios. To see how sensitive our results are to the composition of the asset portfolio we can compare these figures with Fig. 4.

When the equity holdings in the portfolio increase, we find a similar trend as in the base case. The changes in allocation of the reduced total SCR are again small for $\alpha =99.5\%$, i.e., using VaR or ES leads to similar stress scenarios for all three risk classes. When the quantile $\alpha$ decreases, this difference becomes more significant and is largest for $\alpha \approx 98.5\%$. For this quantile, the longevity SCR and the interest rate SCR are larger and the equity SCR is smaller when the SCR is based on ES. When we decrease the value $\alpha$ even more until 97.5%, the differences are getting smaller.

Figure 11 displays a similar trend as the base case, except for one difference. The similarity with the base case is that the change in allocation of the reduced total SCR is small for the quantile $\alpha =99.5\%$, is largest for the quantile $\alpha \approx 98.5\%$, and if we decrease the quantile $\alpha$ until 97.5% the differences are getting smaller again. This figure differs from the base case if we consider the percentage change in SCR per risk class. When an asset portfolio of 10% equity and 90% bonds is used, the largest percentage change is for the equity SCR. Whereas the relative change in equity SCR grows in comparison with the base case, the change in longevity SCR and interest rate SCR decreases in comparison with the base case. Overall, we still conclude that the longevity SCR is larger and the equity SCR is smaller when ES is used.

Figure 12 looks somewhat different. In this case, the asset portfolio consists of 100% in bonds, so that there is no equity risk. We see however that the interest rate SCR is smaller for all quantiles when ES is used.

From comparing Figs. 4, 10 and 11, we get that for different asset portfolios it still holds that the differences in the SCR allocation are largest at $\alpha \approx 98.5\%$ and that the longevity SCR is larger and the equity SCR is smaller when ES is used. The percentage change per risk class differs if the equity holdings change in the portfolio, but the main trends remain similar.

#### Changing the liability portfolio

To test if our results are sensitive to the composition of the liability portfolio, we vary with the distribution of the policyholders and compare the allocation of SCR($\textit{VaR}_{\alpha }$) with the allocation of SCR($\textit{ES}_{\theta (\alpha )}$). We show this comparison for the following liability portfolios:

- young life annuity insurer: policyholders with an average age of 35;
- base case life annuity insurer: policyholders with an average age of 50;
- old life annuity insurer: policyholders with an average age of 65.

All other assumptions made in Sect. 4 still hold. For a precise description of the liability portfolios, see Table 7 in Appendix 1. The number of policyholders per liability portfolio is chosen based on matching the BEL to the BEL of the base case. Similar to the base case, the asset portfolio consists of 25% in equity and 75% in bonds. We again match $37.5\%$ of the duration of the liabilities to determine the amounts invested in the 5- and 30-year bonds.

We find that for the old life annuity insurer, the reduced total SCR is relatively large compared to the BEL. The value of SCR($\textit{VaR}_{99.5\%}$) is 20.98% of the BEL, compared to 23.24% for the baseline company. However, the longevity SCR for the old company is 5.53%, and so larger than for the baseline company. The interest rate SCR for the old company is however 6.42%, which is much smaller than 10.05% for the baseline company. So, interest rate risk dominates the effect on the total SCR. These findings for the young life annuity insurer are qualitatively similar.

**Fig. 13:**
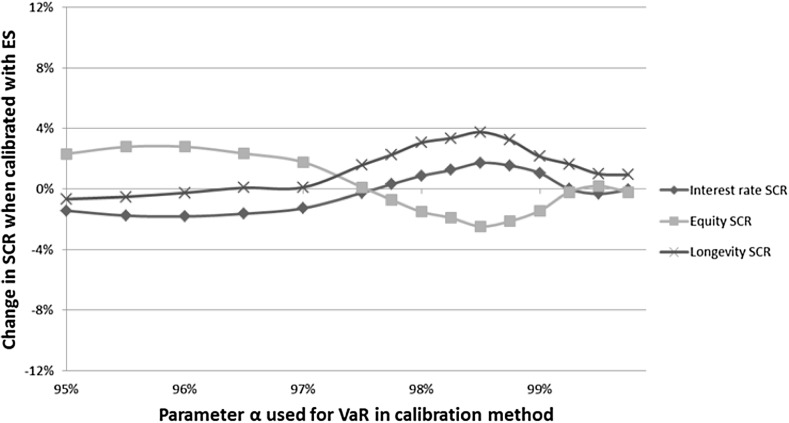
Comparing the allocation of SCR($\textit{VaR}_{\alpha }$) with the allocation of SCR($\textit{ES}_{\theta (\alpha )}$) for the young life annuity insurer. The horizontal axis represents the quantile $\alpha$ used for VaR in the calibration method. The vertical axis represents the change in allocation of the SCRs when the stress scenarios are calibrated with ES instead of VaR

**Fig. 14:**
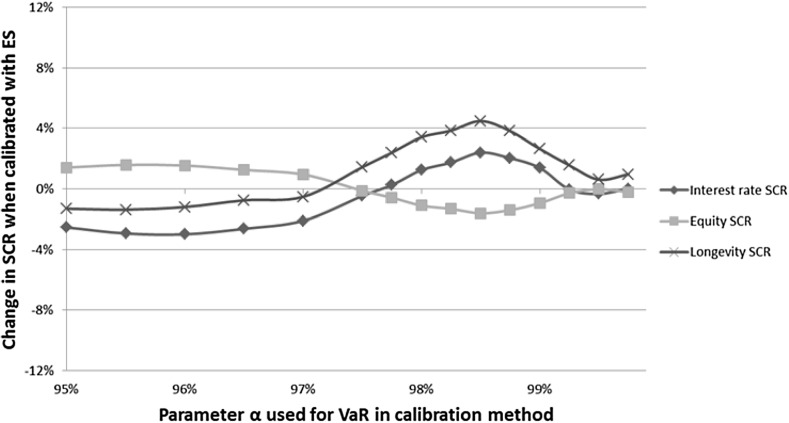
Comparing the allocation of SCR($\textit{VaR}_{\alpha }$) with the allocation of SCR($\textit{ES}_{\theta (\alpha )}$) for the old life annuity insurer. The horizontal axis represents the quantile $\alpha$ used for VaR in the calibration method. The vertical axis represents the change in allocation of the SCRs when the stress scenarios are calibrated with ES instead of VaR

Figures 13 and 14 in Appendix 3 show the change in allocation of the reduced total SCR for the three different risk classes when ES is used to calibrate the shock scenarios instead of VaR for the different liability portfolios. To see how sensitive our results are to the composition of the liability portfolio we can compare Figs. 13 and 14 with Fig. 4. For all liability portfolios, the change in allocation of the reduced total SCR is small when $\alpha =99.5\%$. This difference is largest for $\alpha \approx 98.5\%$; for smaller $\alpha$ the differences are getting smaller until approximately $97.5\%$. We observe that for all liability portfolios that the longevity SCR is larger and the equity SCR is smaller when ES is used. For the value $\alpha =98.5\%$, it holds for all three different liability portfolios that the longevity SCR increases with approximately 4% and the equity SCR decreases with approximately $2.5\%$ if ES was used instead of VaR. If we compare Figs. 13 and 14 we get that relative interest rate SCR changes are amplified for an older composition of the liability portfolio.

## Other regulatory frameworks

The Swiss Solvency Test (SST) is a regulatory framework for insurance companies in Switzerland. The SST uses a holistic approach by taking all risks into the capital requirement calculations, while solvency II has a modular approach. Moreover, the capital requirements in the SST are calibrated using the ES with confidence level 99%.

The differences in SCR($\textit{ES}_{99\%}$) and SCR($\textit{VaR}_{99.5\%}$) are displayed in Table 6. All other assumptions are as in the base case in Sect. 4. We here ignore all other differences of the SST regulation. The reduced total SCR increases from approximately $23.24\%$ of the BEL under the EIOPA standard model to a reduced total SCR determined by $\textit{ES}_{99\%}$ equal to approximately $23.73\%$ of the BEL.

**Table 6:** Overview of the SCR changes of different risk classes, if we switch from using $\textit{VaR}_{99.5\%}$ to $\textit{ES}_{99\%}$

Change reduced total SCR	$2.14\%$
Change interest rate SCR	$3.14\%$
Change equity SCR	$1.37\%$
Change market SCR	$2.03\%$
Change longevity SCR	$3.36\%$

All changes in SCR are expressed in percentage of the BEL

From Table 6, we get that the difference in the reduced total SCR is substantial if $\textit{ES}_{99\%}$ is used. This difference is driven by increases in SCR for all three risk modules.

The Basel III framework is a global regulatory framework for banks which is planned to be implemented in 2019. Basel III was set up in a different manner than solvency II since it is a regulatory framework for a different part of the financial industry. The regulation uses however also stress scenarios to see the impact of shocks on certain risk drivers. The current Basel II framework uses stress scenarios calibrated on value-at-risk with quantile $\alpha =99\%$, but the Basel III framework will be calibrated using expected shortfall with parameter $\theta =97.5\%$. The parameter $\theta =97.5\%$ of ES is set such that the ES corresponds approximately to the $\textit{VaR}_{99\%}$ if the returns are Gaussian. Our analysis for the classes interest rate risk and equity risk suggests that $ES_{97.5\%}$ might lead to lower capital requirements than $VaR_{99\%}$, since $\theta (99\%)=97.81\%$ and $\text {SCR}_{life}(VaR_{99\%})<\text {SCR}_{life}(ES_{\theta (99\%)})$. Note that longevity risk is much less severe for banks. The Basel Committee on Banking Supervision acknowledged the incoherence of the VaR as a risk measurement [6].

## Conclusion

This paper examines the consequences for a life annuity insurer if the solvency II SCR calibration is based on expected shortfall (ES) instead of value-at-risk (VaR). First, we calibrate the SCR stress scenarios for equity risk, interest rate risk and longevity risk based on value-at-risk and expected shortfall. Thereafter, we compare the SCR($\textit{VaR}_{\alpha }$) with the SCR($\textit{ES}_{\theta (\alpha )}$) for a fictitious life annuity insurer.

Since we define $\theta (\alpha )$ such that SCR($\textit{VaR}_{\alpha }$) equals SCR($\textit{ES}_{\theta (\alpha )}$), we focus on the allocation of the reduced total SCR for the three risk classes: equity SCR, interest rate SCR and longevity SCR. For the quantile $\alpha =99.5\%$, as used in solvency II, the difference in allocation is small between SCR($\textit{VaR}_{\alpha }$) with the SCR($\textit{ES}_{\theta (\alpha )}$), i.e., the equity SCR, interest rate SCR and longevity SCR differ little is we use the VaR or ES. Of course, this only applies if the confidence level $\theta (99.5\%)=98.78\%$ is applied for determining the expected shortfall. When $\alpha \approx 98.5\%$, the differences are largest. If we use the ES instead of the VaR to determine the shocks, the longevity SCR is larger and equity SCR is smaller. For smaller values of $\alpha$, the differences become smaller.

These results are robust in terms of variation with the composition of the asset and liability portfolio of the life annuity insurer. To test the sensitivity of our results to the calibration methods used by EIOPA, we compare the results with the SCR allocations when the stress scenarios are determined empirically. We find that when empirical stress scenarios are used, the reduced total SCR increases due to a higher longevity SCR. Moreover, the interest rate SCR is smaller compared to when the calibration methods are as determined by EIOPA [25].

## Figures and Tables

**Fig. 8:**
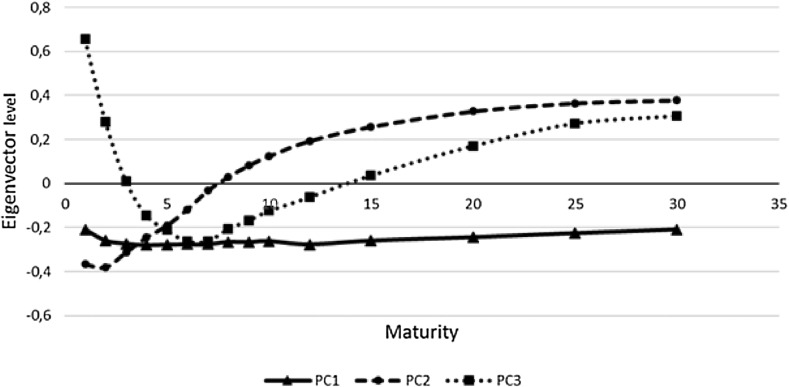
The shift (PC1), twist (PC2) and butterfly (PC3) components of the yield curve of the Euro vs Euribor IR swap rates. The horizontal axis represents the term to maturity and the vertical axis represents the level of the eigenvector component

## Appendix 1: data

See Table 7 and Fig. 7

**Table 7:** The liability portfolio of the young, base case, and old life annuity insurer corresponding to Sects. 4.1 and 5.2

Age	Number of policies Young	Number of policies Base case	Number of policies Old	Accrued yearly payment Starting at age 65
21	7634	361	0	0.067
23	9141	531	0	0.109
25	10646	762	0	0.152
27	12061	1063	0	0.194
29	13289	1441	0	0.236
31	14242	1902	88	0.279
33	14847	2440	138	0.321
35	15054	3046	213	0.364
37	14847	3698	318	0.406
39	14242	4367	462	0.448
41	13289	5016	654	0.491
43	12061	5605	899	0.533
45	10646	6090	1203	0.576
47	9141	6437	1565	0.618
49	7634	6618	1981	0.661
51	6202	6618	2438	0.703
53	4900	6437	2919	0.745
55	3766	6090	3400	0.788
57	2815	5605	3852	0.830
59	2047	5016	4244	0.873
61	1448	4367	4548	0.915
63	996	3698	4742	0.958
65	666	3046	4808	1
67	434	2440	4742	1
69	274	1902	4548	1
71	169	1441	4244	1
73	101	1063	3852	1
75	59	762	3400	1
77	33	531	2919	1
79	18	361	2438	1
81	0	0	1981	1
83	0	0	1565	1
85	0	0	1203	1
87	0	0	899	1
89	0	0	654	1

We assume that all policyholders are born on January 1st

## Appendix 2: interest rate risk stress scenarios by using principal component analysis

In this appendix, we describe the principal component analysis (PCA) to calibrate the interest rate shocks. PCA is used to describe movements of the yield curve and is explained by Fiori and Iannotti [27] and Barber and Copper [5]. It applies an orthogonal linear transformation that converts interest rate changes data of correlated variables into a set of values of linearly uncorrelated variables. For the *m* maturities, the relative yield curve change can then be represented exactly as a linear combination of *m* vectors:

13 $$\begin{aligned} \textit{X}_{t} = c_t+ \textit{b}_{1,t} \textit{U}_{1} + \cdots + \textit{b}_{m,t} \textit{U}_{m} \end{aligned}$$

where $\textit{X}_{t}$ is the relative change in the annualized yield curve at time $t=1,\ldots ,T$, $c_t$ is a time-varying constant, and $\textit{U}_{k}$ are time-independent, zero-mean $m\times 1$ vectors that are orthogonal.

The data is then transformed into a new coordinate system such that the first coordinate (i.e., the principal component $\textit{b}_{1,t}$) explains the largest share of the variance by any projection of the data, the second coordinate has the second largest explanation of the variance, etcetera. The objective of PCA is to determine a small set of components that best explain the total variance of the data. The number of components is then small $K\ll m$, but with high explanatory power:

$$\begin{aligned} \textit{X}_{t} = c_t+ \textit{b}_{1,t} \textit{U}_{1} + \cdots + \textit{b}_{K,t} \textit{U}_{K} + \varepsilon _{t} \end{aligned}$$

where $\varepsilon _{t}$ is a zero-mean error term.

When PCA is used to describe interest rate movements, the factors $\textit{U}_{k},k=1,\ldots ,K$ are the eigenvectors of the covariance matrix of the original data. The first six components explain between 99.2 and 99.5% of the variance with a 90% confidence interval (cf. [5]). The principal components describe the different yield curve movements and the first three are interpreted as the shift, twist and butterfly moves of the yield curve [31, 37]. Figure 8 shows the first three eigenvectors of the Euro vs Euribor IR swap rates. In this figure, we observe the shift, twist and butterfly moves.

The principal components are derived via the following matrix multiplication:

14 $$\begin{aligned} b_{k,t} = U_{k}' X_{t}, \text {for}\,\,k = 1,2,\ldots ,K. \end{aligned}$$

To transform the principal components and eigenvectors into VaR and ES based interests rate stress scenarios, we use a method of Fiori and Iannotti [27]. For a maturity *k*, we regress the relative annual percentage rate changes on the principal components to derive the “beta” sensitivity of each rate to each principal component via (14). We use the empirical distribution of each principal component vector $b_k$. Via historical simulation of the principal components, we derive a down risk stress scenario and an up risk stress scenario for both VaR and ES as follows:

15 $$\begin{aligned} VaR_{\gamma }&=VaR_\gamma \left( \sum _{k=1}^K \hat{b}_{k} \textit{U}_{k}\right) , \text {for}\,\,\gamma = \alpha , (1-\alpha ); \end{aligned}$$

16 $$\begin{aligned} ES_\gamma&=ES_\gamma \left( \sum _{k=1}^K \hat{b}_{k} \textit{U}_{k}\right) , \text{for}\,\,\gamma = \theta (\alpha ),1-\theta (\alpha ). \end{aligned}$$

Since the eigenvectors $U_k,k=1,\ldots ,K$ are orthogonal, we can randomly draw the marginal distributions of $\hat{b}_k,k=1,\ldots , K$ and determine the empirical distribution of $\sum _{k=1}^K \hat{b}_{k} \textit{U}_{k}$ accordingly (see, e.g., [27]). We use 4000 simulations for each dataset.

In our four datasets, we set $K=3$ or $K=4$, which is determined such that the principal components describe at least 95% of the variance. Since we have four datasets, we have four different up and down shock vectors. In line with [25], we set the up and down shock at maturity of 90 years and longer at $+$ 20 and − 20%. Moreover, the swap rates are not always defined for every maturity, and therefore linear interpolation is used to fill in shocks for these maturities. The mean result of the four different up and down shock vectors has been taken to arrive at a generalized up and down shock vector [26].

## Appendix 3: figures sensitivity analysis

See Figs. 9, 10, 11, 12, 13 and 14.

## Footnotes

1 As shown by Yamai and Yoshiba [46], it holds that if the random variable *X* is Gaussian with mean $\mu$ and standard deviation $\sigma$, then $\textit{VaR}_\alpha (X)=\mu +q_\alpha \sigma$ and $\textit{ES}_\theta (X)=\mu +[\phi (q_\theta )/(1-\theta )]\sigma$, where $q_\alpha$ is the quantile-function of a standard Gaussian distribution, and $\phi$ is the density function of a standard Gaussian distribution.

2 Adding a risk margin (based on the total SCR) would lead to a circular argument. This is noted by Coppola and D’Amato [15].

3 See http://www.toezicht.dnb.nl/en/binaries/51-226788.pdf (last accessed: November 9th, 2016) or [13] for more information on the procedure to calibrate the interest rate term structure.

4 For EEA government bonds that have a AAA credit rating, an insurer is not required to hold an SCR for spread risk by EIOPA [21].

5 The coupon rates are based on the 5- and 30-year UK Gilt bonds on December 31st, 2014.

6 For the detailed mortality table that we use, see http://www.ag-ai.nl/download/14127-Prognosetafel+AG2012-2062.xls (last accessed: May 4th, 2017).

7 If all returns were Gaussian, we would have $\theta (99.5\%)\approx 98.71\%$. A commonly used rule of thumb is that $\textit{VaR}_{\alpha }=\textit{ES}_{\theta }$, where $\theta \approx 1-2.5(1-\alpha )$ for large values of $\alpha$. The Basel III regulation moves for instance from a VaR with $\alpha =99\%$ to an ES with $\theta =97.5\%$. In Fig. 2, we see that this relation holds for smaller quantiles $\alpha$, but the factor 2 (instead of 2.5) provides a better representation for large quantiles. So, if an ES with $\theta =99\%$ is used instead of a VaR with $\alpha =99.5\%$, the reduced total SCR increases.

8 See https://www.ecb.europa.eu/stats/services/sdmx/html/index.en.html (last accessed: May 4th, 2017).

9 See http://www.bankofengland.co.uk/statistics/yieldcurve/archive.htm (last accessed: May 4th, 2017).

10 If the fictitious life annuity insurer applies duration matching, we still find that the down-shock is more harmful than the up-shock.
